# Dual Intra-Articular Fracture of the Body and Hook of Hamate: A Delayed Presentation

**DOI:** 10.1155/2022/6774826

**Published:** 2022-02-14

**Authors:** Eimear Phoenix, John Mahon, Robert M. Kenyon, Rafal Pajda

**Affiliations:** Midland Regional Hospital Tullamore, Tullamore, Offaly, Ireland

## Abstract

Fractures of the hamate bone are an unusual clinical entity. Dual fractures involving both the body and the hook of the hamate are even more unique, with only two previous cases described in the literature, to our knowledge. Clinicians are often unfamiliar with the presenting signs of this unusual injury, and subsequently, diagnosis is often delayed or missed entirely. We describe the case of a 19-year-old male who sustained an intra-articular body of hamate fracture with an ipsilateral hook of hamate fracture in his dominant hand. He presented 10 days following the initial injury and was managed with open reduction and internal fixation (ORIF).

## 1. Introduction

Isolated carpal fractures are uncommon. Solitary hamate fractures are particularly rare, accounting for 2-4% of all carpal bone fractures [1]. Due to the intricate architecture of the carpal bones, physicians may be unfamiliar with the standard anatomy, making pathological diagnoses challenging.

Overlooking this unique bony injury can result in mal- or nonunion and a progressive sequela of osteoarthritis and chronic pain [2].

This report is aimed at increasing awareness of hamate fractures and their patterns of injury. The authors highlight the importance of maintaining a high level of clinical suspicion in the context of traumatic ulnar-sided wrist pain, to ensure timely diagnosis and appropriate management of these uncommon injuries.

## 2. Case Presentation

We, the authors, present the case of a healthy 19-year-old right hand-dominant male who presented to our emergency department (ED) with pain and swelling in his right wrist. His clinical history was significant for trauma to his right hand, having punched a plank of wood whilst intoxicated 10 days prior to presenting to ED. Apart from a 1 pack-year smoking history, our patient had no medical comorbidities and was unemployed.

On clinical examination, there was generalized tenderness and mild oedema over the right wrist joint, with maximum tenderness over the volar ulnar aspect. Range of motion of the wrist was reduced secondary to pain, with maximum discomfort on wrist flexion. There was no clinical evidence of neurovascular deficit, and grip strength was not compromised. A deformity was noted over the dorsal aspect of the right 5^th^ metacarpophalangeal (MCP) joint which the patient reports as longstanding from a previous injury; examination of the remaining MCP joints and interphalangeal (IP) joints was otherwise normal. There was no tenderness over the anatomical snuffbox, and the proximal limb was unremarkable.

Given the clinical concern for bony injury, an X-ray of the right wrist was performed including posteroanterior and oblique views. Carpal tunnel and specialised lateral views were omitted due to the patient's limited range of motion secondary to pain.

**Figure 1:**
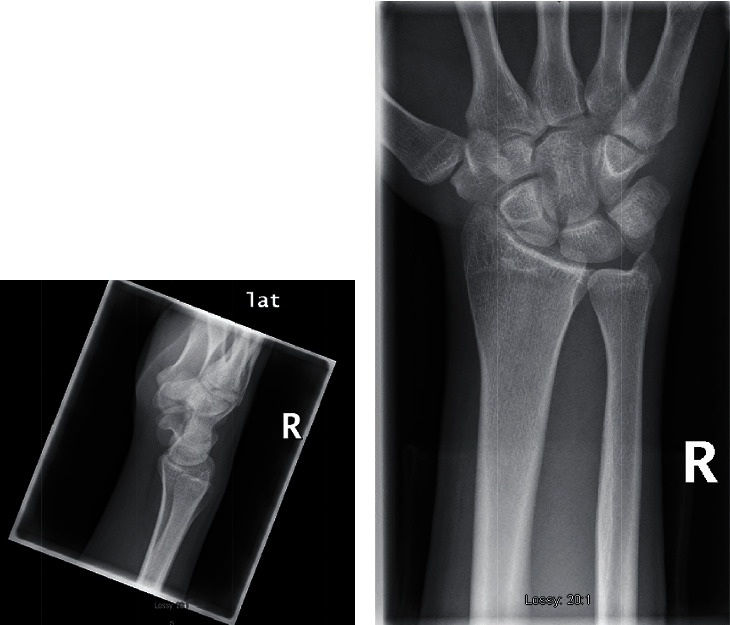
(a, b) X-ray right wrist PA and lateral views suggestive of a dual hamate fracture.

**Figure 2:**
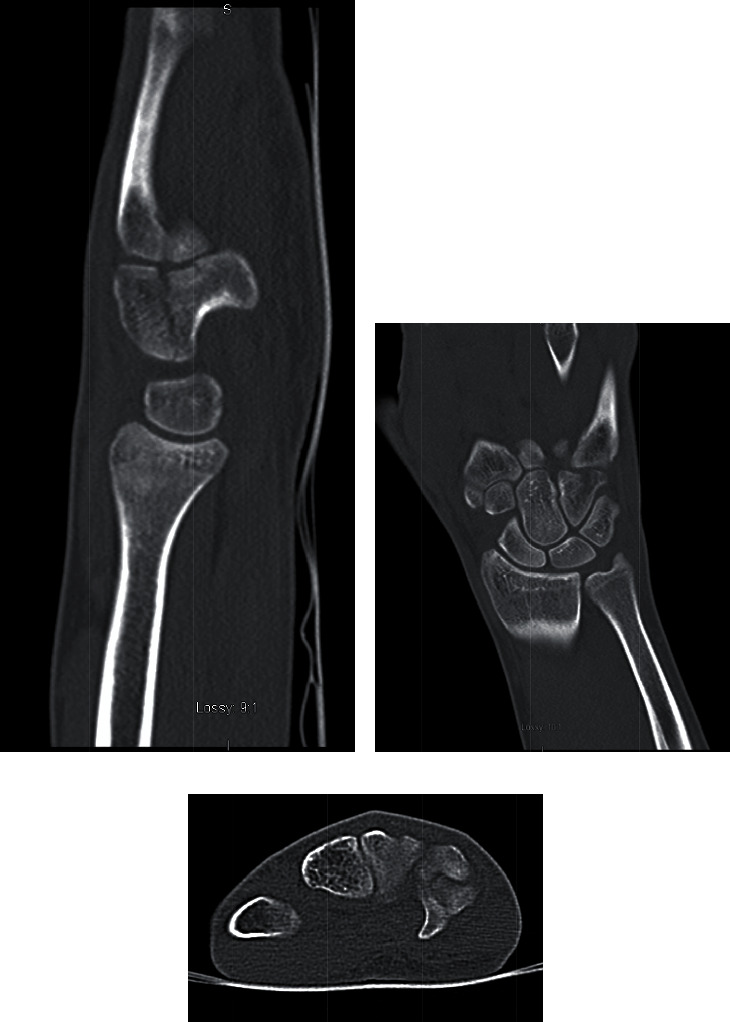
(a–c) CT images showing a longitudinal split fracture of both the hook and body of the hamate with 2 mm of dorsal displacement and intra-articular extension involving the CMC joint.

Plain films were suggestive of a dual hamate fracture (Figures 1(a) and 1(b)), and a computed tomography (CT) of the right wrist was performed to confirm diagnosis and to further characterize the bony injury (Figures 2(a)–2(c)). The CT confirmed a longitudinal split fracture of both the hook and body of the hamate with 2 mm of dorsal displacement and intra-articular extension involving the carpometacarpal (CMC) joint (Figures 1(a)–1(c)).

The following day, the patient underwent open reduction and internal screw fixation of the body and hook of hamate by consultant orthopaedic hand surgeon and senior author RP. A K-wire was used to stabilise the 3^rd^-5^th^ metacarpals, and a Futura splint was applied to be worn 24/7 until outpatient review.

**Figure 3:**
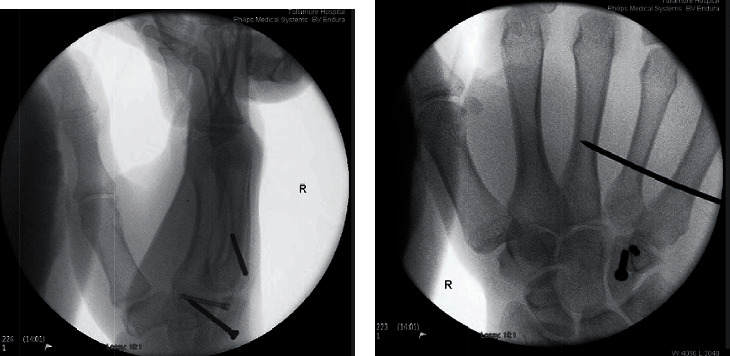
(a, b) Intraoperative screening showing satisfactory reduction and alignment of right dual hamate fracture using screw fixation and K-wire stabilisation to the 3^rd^-5^th^ metacarpals.

Peri- and postoperative films showed satisfactory reduction and alignment (Figures 3(a) and 3(b)). The patient's immediate postoperative course was uneventful, neurovascular status was normal, and pain was well controlled. The importance of abstaining from smoking to ensure adequate healing was reinforced prior to discharge.

**Figure 4:**
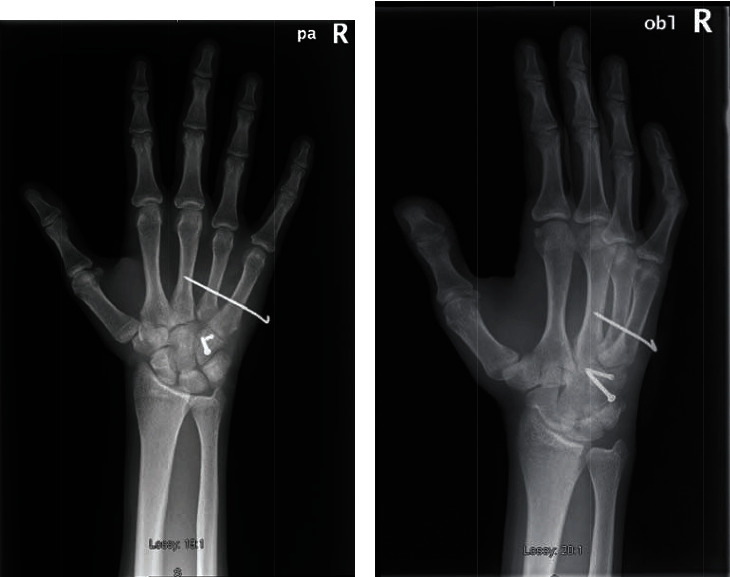
(a, b) X-ray right hand PA and oblique 4 weeks postoperatively demonstrating satisfactory position, alignment, and evidence of healing.

The patient was reviewed 1 week postoperatively at our dressing clinic; his wound was healing well with no evidence of superficial infection. He attended clinic 4 weeks postoperatively; repeat X-rays showed stable fixation and satisfactory alignment of the carpal arch (Figures 4(a) and 4(b)), and the stabilising K-wire was removed. His wound was well healed, neurovascular status was intact, and there was no tenderness elicited on examination. Grip strength was recorded as 21.8 kg (right hand) and 34.5 kg (left hand), and he was referred for hand therapy with a plan for outpatient review in a further 2 months' time. Unfortunately, our patient did not attend for further follow-up on two occasions and was next reviewed 1 year following his initial injury. At 1 year follow-up, he remained pain free and had no functional deficit. Range of motion was satisfactory with 70 degrees wrist flexion bilaterally and wrist extension 70 degrees (left) and 60 degrees (right). Dynamometry demonstrated a grip strength of 41 kg (left) and 39.6 kg (right), and he was subsequently discharged from our service.

## 3. Discussion

Hamate fractures are unusual and pose considerable diagnostic and therapeutic challenges.

The hamate is located at the ulnar aspect of the distal carpal row, a wedge-shaped bone with a curved prominence, namely, the hamulus or “hook” [1]. Milch classified hamate fractures into two major groups: the hook (type 1) and the body (type 2) [3].

The hook is the most commonly fractured part of the hamate bone [3]. Extending from the palmar aspect of the body, it establishes the medial boundary of the carpal tunnel and lateral aspect of Guyon's canal [4]. The hook of hamate attaches various ligaments and tendons to serve as a pulley for the flexor tendons of the 4^th^ and 5^th^ digits [4].

Mechanism of injury of concurrent fractures of the body and hook is postulated to be a combination of direct and indirect forces resulting in ulnar axial injury [1–4].

Such as in our patient's case, injury to the hamate body commonly occurs when a clenched fist strikes a solid surface. First impact occurs axial to the forearm, encountering the clenched fist which transmits the impact through the 4^th^ and 5^th^ metacarpals to the body of hamate, followed by indirect traction and avulsion of the hook [1–4].

Fractures of the hamate bone occur more frequently amongst athletes, particularly racquet games, baseball, and golf [5]. In this context, hamate fractures result from incorrect positioning of equipment such as a racquet handle or end of a golf club, leading to transmission of force directly through the carpal arch [5]. Alternatively, when catching a propelled object, such as a baseball, shearing force from the 4^th^ and 5^th^ flexor tendons travels through the hamulus and may result in a fracture [6].

Physical examination of the hamate bone can be challenging to interpret. Clinicians are often unfamiliar with the anatomy of the carpal arch and superimposed hypothenar tissues, leading to misdiagnoses and a subsequent delay in management [1].

The pisiform bone neighbours the hamate and acts as a useful landmark to guide clinical examination. The hamate can be more clearly palpated along the line extending from the pisiform to the 3^rd^ metacarpal head [3, 4].

Presentation can be variable, reported cases describe minimal swelling, and point tenderness elicited over the dorsal aspect of the hamate and pain whilst gripping an object or during supination and pronation of the wrist [7].

Radiographic investigations are an essential adjuvant to diagnosing and further characterizing hamate fractures. Plain radiographs are typically the first investigation performed in the emergency department for suspected bony injuries [8]. Hamate fractures seldom present with a clear fracture lucency on plain film, and many centres may omit more informative views such as oblique, ulnar-deviated posteroanterior and carpal tunnel [8, 9]. Ebraheim et al. reported 11 cases of hamate fractures; routine plain film X-ray failed to identify a hamate fracture in 5 of these patients, leading to an average delay in presentation of 10 days [10]. A high level of clinical suspicion must be maintained if a carpal fracture is suspected, particularly when initial X-rays are inconclusive.

Plain film X-ray has a sensitivity of 72.2% and specificity of 88.8%, inferior to that of high-resolution computed tomography (HR-CT) with a reported sensitivity 100% and specificity 94.4% [11]. Subsequently, HR-CT is considered the diagnostic imaging modality of choice and is also important in further characterizing the bony injury to guide management. MRI is of beneficial if there is concern regarding ligamentous or tendinous injuries [11].

Complications of hamate fractures are not uncommon, particularly in cases of delayed diagnosis. Overlooked pathology and misdiagnoses can result in non- or malunion and a progressive sequela of osteoarthritis, chronic pain, and potential dysfunction [4, 12, 13]. Insult to surrounding anatomy can result in rupture of the flexor digitorum profundus (FDP) tendons or nerve compression, including the ulnar nerve [14]. The deep motor branch of the ulnar nerve runs along the base of the hook whilst the superficial sensory branch is closely related to the tip of the hamulus [14]. Due to the structural relationship of the hamate with the ulnar aspect of the carpal tunnel, insult to the hamate may cause dislocation towards the tunnel resulting in median nerve compression [14, 15]. This is demonstrated by Manske who described a case of a fractured hook of hamate presenting with symptoms of carpal tunnel syndrome [15].

Unlike scaphoid fractures, avascular necrosis is fortunately a rare event with hamate fractures, owing to its triple vascular supply [16].

Management aims of hamate fractures are stabilisation of the articular surface of the distal hamate and maintaining function of the 4^th^ and 5^th^ CMC joints [7, 17].

Stable fractures without articular involvement can be managed by closed reduction and cast immobilisation or splinting, with consideration for any related soft tissue injury [13].

Surgical management is advocated for displaced fractures or articular fractures involving more than 1/3 of the CMC articular surface [7].

Dual fractures involving both the body and hook of hamate are inherently unstable and require surgical fixation [17]. Isolated hooks of hamate fractures are typically managed conservatively with consideration of delayed excision of the fracture fragment in patients that remain symptomatic beyond 6 weeks [17].

Given the intrinsic instability of the fracture configuration in our patient's case, we felt it was optimal to address the hook of hamate fracture concurrently during fixation of the body of hamate fracture. Given the size and orientation of the hook fragment, the authors agreed that it was better suited to compression screw fixation rather than excision.

In 2005, Kapickis et al. reported a case of a combined left hook and body of hamate fracture of a left hand-dominant male, who successfully managed with ORIF [7]. In 2012, Arora et al. reported a second case of dual fractures of the hamate, this time in the nondominant hand of a 28-year-old male, which was managed with closed reduction and internal fixation using percutaneous pinning of the hamate body only [17].

Due to the nature of injury, carpal bone fractures usually occur in the patient's dominant hand, as in our patient's case. Restoring and maintaining function is paramount, particularly given the usual cohort of patients who present with these injuries and young males of working age with a severe injury to their dominant hand.

## 4. Conclusion

Dual fractures of the body and hook of hamate are exceedingly rare. With increased popularity of racket sports, the incidence of carpal bone fractures is likely to increase. Physician unfamiliarity and discrepancy in presentation are complicated by sophisticated anatomy; this injury is commonly overlooked clinically and underreported radiologically.

The authors present to our knowledge the first case of a delayed presentation of a combined intra-articular body and hook of hamate fracture described in the literature.

We hope that through reporting our experience, this case will raise awareness of hamate fractures including their presentation and potential mechanism of injury. We encourage other clinicians to report these unusual bony injuries if encountered, to help aid timely diagnosis and appropriate management.
